# Simulating and Testing Microvibrations on an Optical Satellite Using Acceleration Sensor-Based Jitter Measurements

**DOI:** 10.3390/s19081797

**Published:** 2019-04-15

**Authors:** Shan-Bo Chen, Ming Xuan, Lei Zhang, Song Gu, Xiao-Xue Gong, Hong-Yu Sun

**Affiliations:** 1 Changchun Institute of Optics, Fine Mechanics and Physics, Chinese Academy of Sciences, Changchun 130033, China; ak48css@sina.com (S.-B.C.); 18686344285@163.com (L.Z.); gusong@charmingglobe.com (S.G.); gxx@mail.ustc.edu.cn (X.-X.G.); 2 University of Chinese Academy of Sciences, Beijing 100049, China; 3 Chang Guang Satellite Technology LTD, Changchun 130033, China; sunhongyu@charmingglobe.com

**Keywords:** microvibration, optical amplification factor, acceleration sensor, jitter measurement, pixel offset

## Abstract

The present study uses a method to address microvibrations effects on an optical satellite by combining simulations and experiments based on high-precision acceleration sensors. The displacement and angular displacement of each optical component can be obtained by introducing flywheel perturbation data from a six-component test bench to the finite element model of the optical satellite. Combined with an optical amplification factor inferred from the linear optical model, the pixel offset of the whole optical system is calculated. A high accuracy and broad frequency range for a new microvibration measurement experimental system is established to validate the simulation. The pixel offset of the whole optical system can be measured by testing the acceleration signals of each optical component and calculating optical amplification factors. The results are consistent with optical imaging test results, indicating correctness of the experimental scheme and the effectiveness of the simulation. The results suggest that the effect of microvibrations on a camera can be verified by using mechanical simulators instead of a whole optical camera for the experiment scheme, which is demonstrated to be an effective way for increasing efficiency in jitter measurements.

## 1. Introduction

Along with the rapid development of Earth observational technology, both the resolution and the pointing accuracy of optical satellites are getting higher. Cameras are becoming more sensitive to microvibrations associated with the active part of the satellite in orbit [1]. To provide altitude control torque or to maintain stability, flywheels are commonly used as altitude control actuators in high-performance spacecrafts [2]. However, it inevitably produces perturbations under working conditions, resulting in decreased pointing accuracy of the satellite and degraded imaging performance [3,4]. Microvibrations are difficult to measure because they have small amplitudes. Judging the effect of perturbations through optical imaging testing seems to be a feasible program [5,6]. However, the harsh application environment and long lead time of space cameras greatly restrict the application of this scheme. Therefore, combining simulations and more reasonable test schemes is of great importance, not only for the evaluation of optical images but also for the application of vibration control measures [7,8].

The effect of microvibrations on optical satellites is complicated, as it includes structure, control, and optics [9,10]. So far, an effective means is to integrate the structure, control, and optical system as a whole calculation model; this reflects the relationship between microvibrations and the camera, from perturbation sources through the loading path to imaging quality [11,12]. The whole calculation model can be checked and verified by ground testing [13]. Validation tests on ground are key in microvibration research. Up to now, two kinds of test methods for optical satellites have been mainly adopted. One is an optical imaging test, which simulates the condition of the camera in orbit to show the influence of microvibrations [14,15]. The result is not only intuitive but also consistent with the camera’s in orbit performance. The other method is by using a high-precision acceleration sensor to measure the mechanical response characteristics of key components. This scheme is mainly used to test the efficiency of microvibration isolation [16].

**Figure 1:**
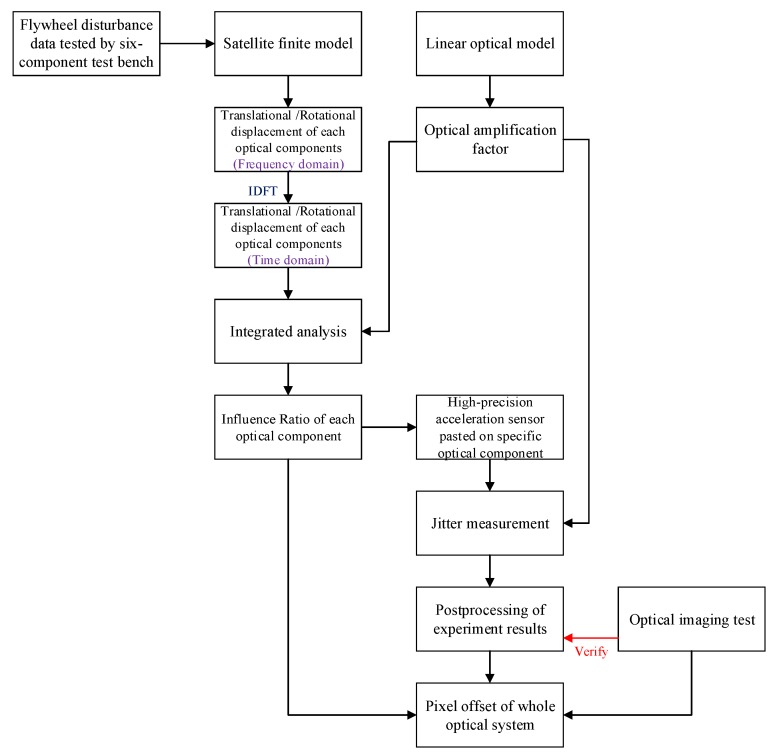
A flow chart showing the combination of the simulation and the experiment.

This paper focuses on the influence of flywheel microvibrations on an optical satellite. Pixel offset can be obtained by simulation and experiments based on a high-precision acceleration sensor. The flow of the analysis and experiment is shown in Figure 1. The displacement and angular displacement of each optical component are calculated in the frequency domain, and the data are transformed into time domain using inverse discrete Fourier transform (IDFT). Pixel offset of the whole optical system can be calculated by integrating the displacement and angular displacement of each optical component with its optical amplification factor, computed by a linear optical model. The simulation scheme can not only predict the pixel offset of the whole camera, but also analyze the effect of perturbation influences on each optical component, which can provide guidance for later experiments [17]. The experiment scheme based on high-precision acceleration sensors can reflect the amplification of microvibrations along a loading path as well as measure pixel offset of the whole optical system by integrating an optical amplification factor.

The paper is organized as follows. In Section 2, the development of a linear optical model is discussed to calculate the optical amplification factor of each optical component. Section 3 analyzes pixel offset of the whole optical system through simulation. The influence ratio of each optical component on pixel offset is calculated. Section 4 discusses the comparison between two test results and the simulation, and this is followed by a list of conclusions in Section 5.

## 2. Optical Model

**Figure 2:**
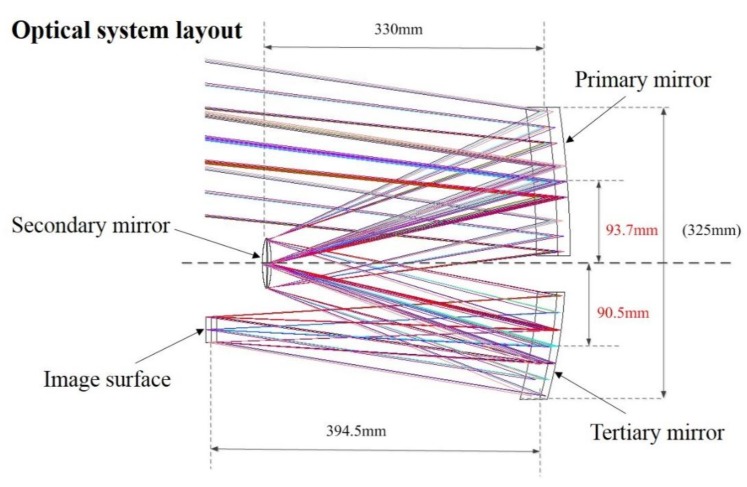
Optical layout of the camera.

The optical system contains four optical components, and the relative position of each optical component is shown in Figure 2.

If the perturbation is small, the deformation of an optical component is negligible, indicating that the optical component can be assumed as a rigid body. The optical model used for jitter analysis can be briefly described as a first-order Taylor expansion of the image motion of a central image point on the focal plane [18,19]. The linear optical model can be expressed as:

(1) $$L=L_{0}+\frac{\partial L}{\partial U}\Delta U+ο(2)$$

where *L* is the image motion of a central image point on the focal plane; *L*_0_ is the nominal decentration of a central image point; ΔU is the perturbation of each degree of freedom (DOF); ο(2) represents the second and higher order terms of the expansion, which is neglected in the linear analysis; and $\frac{\partial L}{\partial U}$ is the optical amplification factor representing the decentration of a central image point on the focal plane associated with each unit transition/tilt of optical component.

Since +*X* is the flight direction of the satellite, decentration on the focal plane is projected to the *X* direction (along the flight direction) and *Y* direction (normal to the flight direction) to calculate pixel offset of the image. The optical amplification factor of each optical component is calculated with optic design software (Zemax) and shown in Table 1.

## 3. Simulation

### 3.1. Testing Flywheel Perturbation

**Figure 3:**
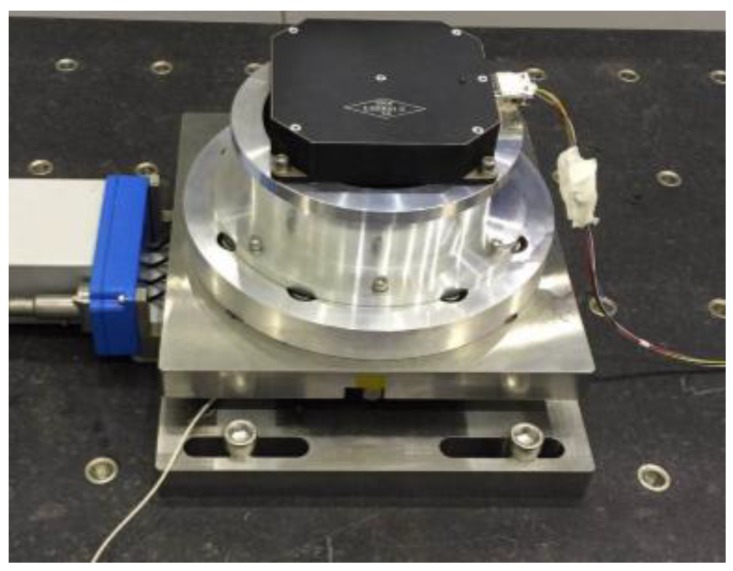
Testing flywheel perturbation.

**Figure 4:**
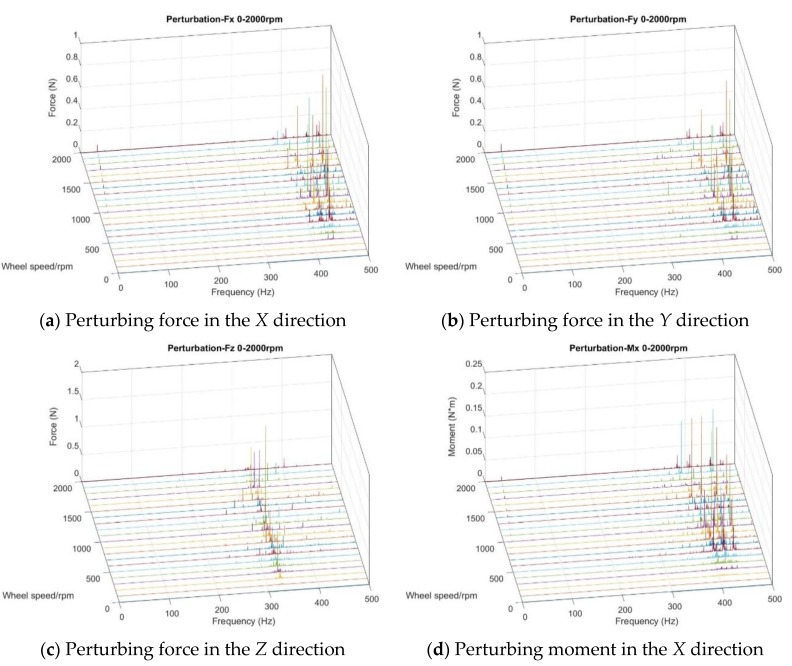

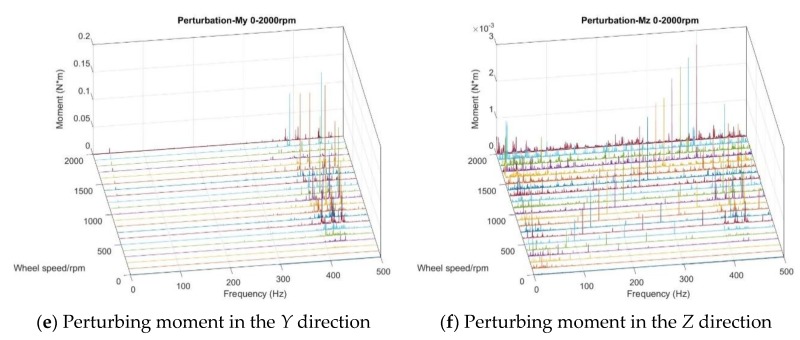
Waterfall plot of perturbations in each degree of freedom (DOF).

The test of flywheel perturbation is performed with a six-component test bench (Kistler table/HR-FP3402) installed in the air bearing a floating platform, as shown in Figure 3. The flywheel is fixed to the test bench through a rigid fixture [20,21]. Under push broom imaging conditions during orbit, the flywheel spins at a constant rotation speed. Therefore, the output data are intercepted under steady state in different rotation speeds with the sampling frequency set to 4096 Hz. In light of the misalignment between the centroid of the flywheel and the center of the test bench, the perturbing force/moment of the flywheel can be calculated in the following equation:

(2) $$\left\{ \begin{array}{l} F_{i}=F_{i}^{t}\\ M_{i}=M_{i}^{t}-\sum \left(F_{i}^{t}\cdot \Delta \right) \end{array}\right.$$

where $F_{i}/M_{i}$ is the perturbing force/moment of the flywheel in each direction (i=1,2,3); $F_{i}^{t}/M_{i}^{t}$ is the measured perturbing force/moment in each direction; and Δ represents the distance from the centroid of the flywheel to the center of the test bench in each direction. The disturbing force/moment of the flywheel is attributed to several reasons, including rotor imbalance, bearing irregularity, flexibility of the flywheel structure, and nonlinear stiffness of bearing [22]. To better understand the characteristics of perturbation, the test data (time domain) are transformed to frequency domain by using fast Fourier transform (FFT) [23]. The response curves of perturbation in each DOF are shown in Figure 4.

As seen from Figure 4, it can be concluded that the perturbation was mainly focused on the first harmonic order because of the rotor imbalance and high frequency (over 300 Hz). The amplitude increased with the rotor speed for the first harmonic order perturbation. Aiming at the lateral perturbation (*X/Y* direction), a V-shape frequency distribution curve was seen around 400–450 Hz because of the rocking whirl mode of flywheel. The perturbing force in the *Z* direction at different speeds was concentrated at 330 Hz because of the lateral mode of the flywheel [24,25].

### 3.2. Integrated Analysis

**Figure 5:**
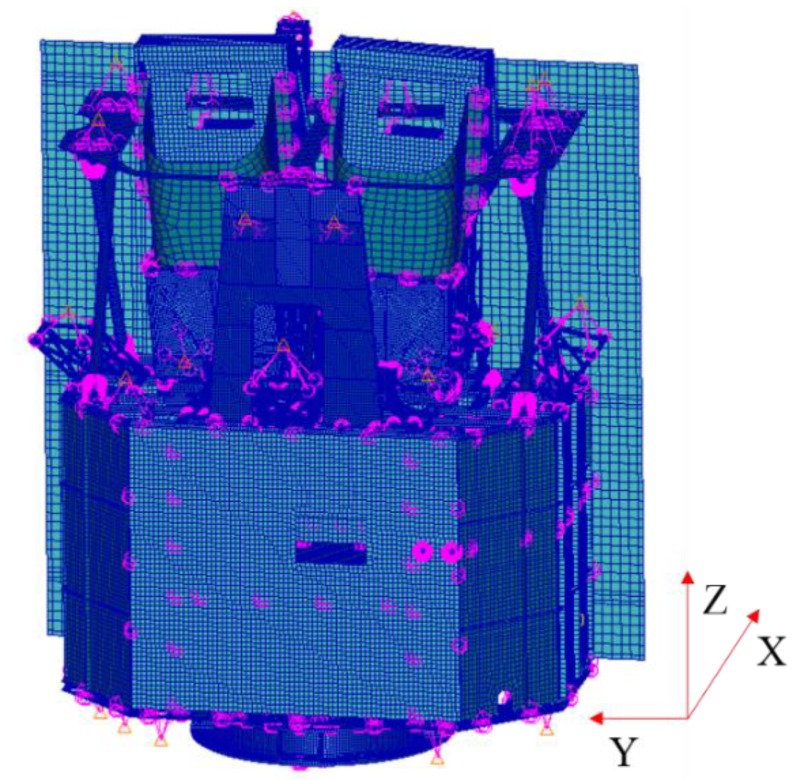
Satellite finite element model (FEM).

**Figure 6:**
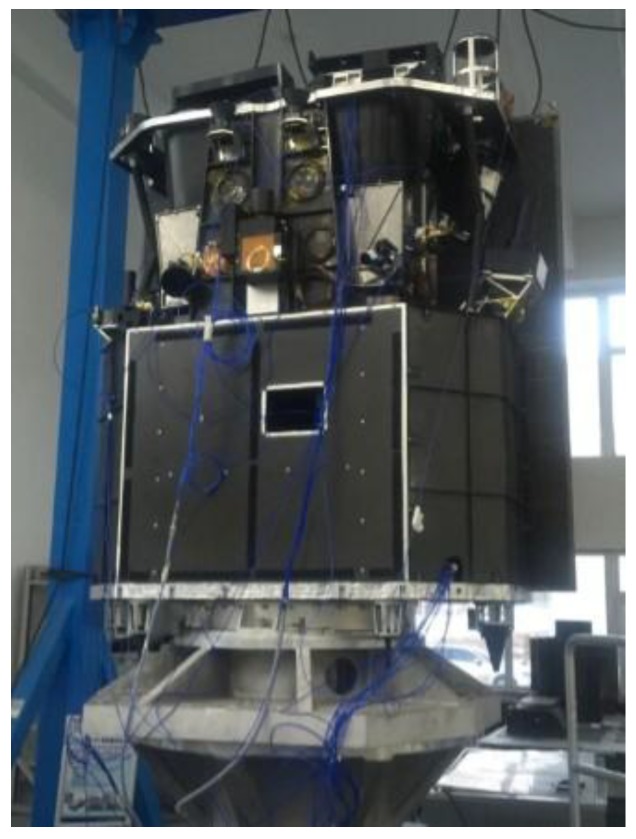
The lift-off vibration test of the satellite.

**Figure 7:**
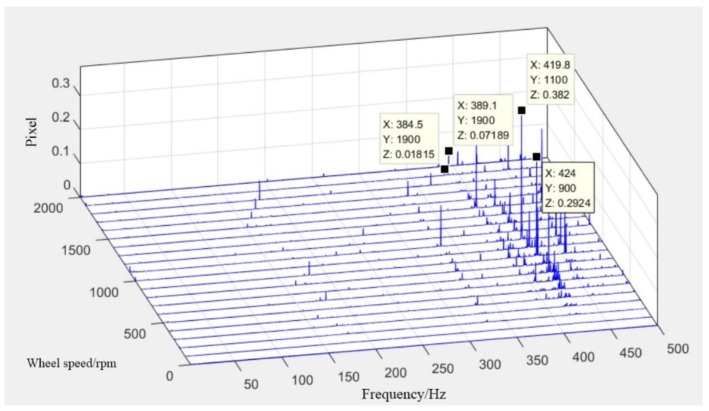
Simulated results of pixel offset vertical to flight direction.

Integrated analysis supports structure design and verification of high-level optical requirements for image quality and sensitivity [26,27,28]. It can also extrapolate the effect of microvibrations on opto-mechanical stability [29]. The finite element model (FEM) of the satellite was established by software (Patran), as shown in Figure 5. The lift-off vibration test was performed to verify the correction of FEM, focusing on the spectrum characteristics of optical components, as shown in Figure 6. The first order natural frequencies of the satellite in the *X/Y/Z* direction were 25, 30, and 135 Hz, respectively. The first order mode shapes in the *X/Y* direction were shown as the horizontal swing, and the first order mode shape in the Z direction was shown as axial translational motion. Moreover, unit force/moment was applied, respectively, at the flywheel mounting position, and the displacements of three specific points on each optical component were obtained. The analysis was performed using nominal 0.1% damping of the critical value in the satellite structure [6]. With the displacements of three specific points, the displacement and angular displacement of each optical component could be calculated by a three-point fitting algorithm. After multiplying the measured perturbation in each DOF by the displacement and angular displacement of the optical component, the results were transformed to time domain by IDFT. Finally, integrated with the optical amplification factor of each optical component and sum, the decentration of the central image point on the focal plane could be calculated by MATLAB processing. The pixel offset normal to the flight direction of the whole optical system after FFT is shown in Figure 7.

As shown in Figure 7, the influence on pixel offset was mainly concentrated at 330 Hz due to axial perturbation and 390–450 Hz due to lateral perturbation, respectively. The maximum pixel offset reached 0.382 pixels at 419.8 Hz.

### 3.3. Influence Ratio of Each Optical Component

**Figure 8:**
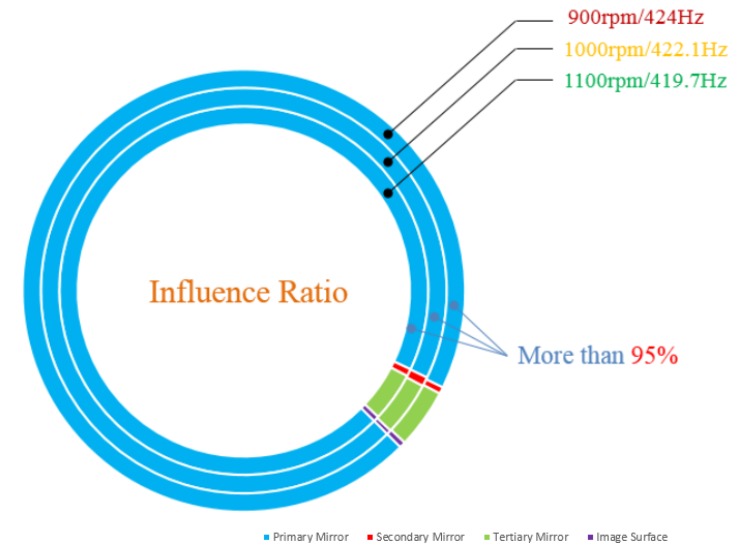
Influence ratio of each optical component.

The simulation scheme could also be used to analyze the influence ratio by disintegrating the pixel offset of the whole optical system to each optical component. The influence ratio could substantially improve the structural design of optical components and arrange a reasonable isolation. The influence ratio of each optical component at different speeds are shown in Figure 8 by extracting the top three peaks. Perturbation had the greatest influence on the primary mirror in this opto-mechanical system when the pixel offset fell below 500 Hz, wherein the influence ratio was over 95%. In contrast, perturbation was independent on the secondary mirror and image surface. Particularly, the sum of two components accounted for no more than 2%.

## 4. Experiment

### 4.1. Test Process

**Figure 9:**
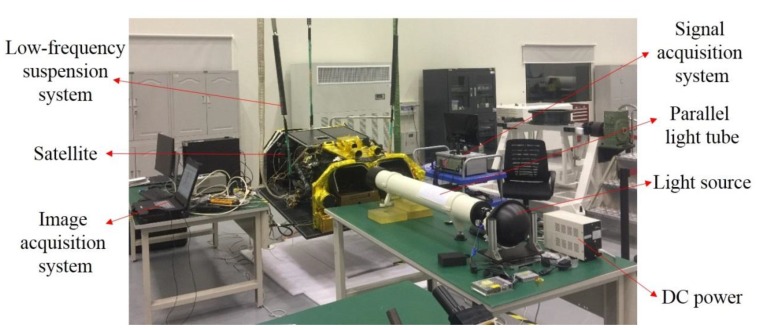
Test platform.

In this paper, the effect of microvibration on image motion was tested based on a high-precision acceleration sensor (PCB, 356B18). The camera imaging test was carried out simultaneously to check and verify the experiment results. The test platform was mainly composed of three parts and has been built to simulate the on-orbit imaging conditions, including a low-frequency suspension system, signal acquisition system, and an image acquisition system, as shown in Figure 9.

**Figure 10:**
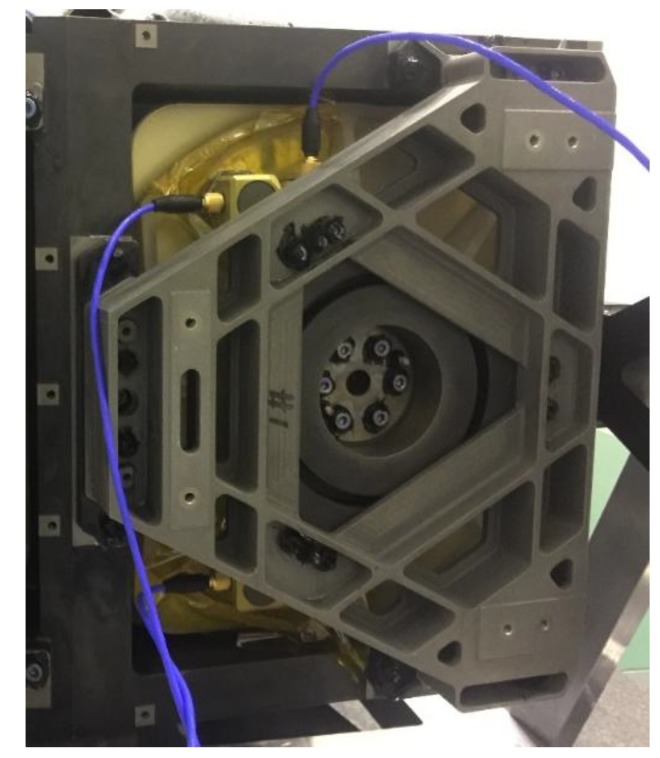
Distribution of acceleration sensors pasted on PM.

**Figure 11:**
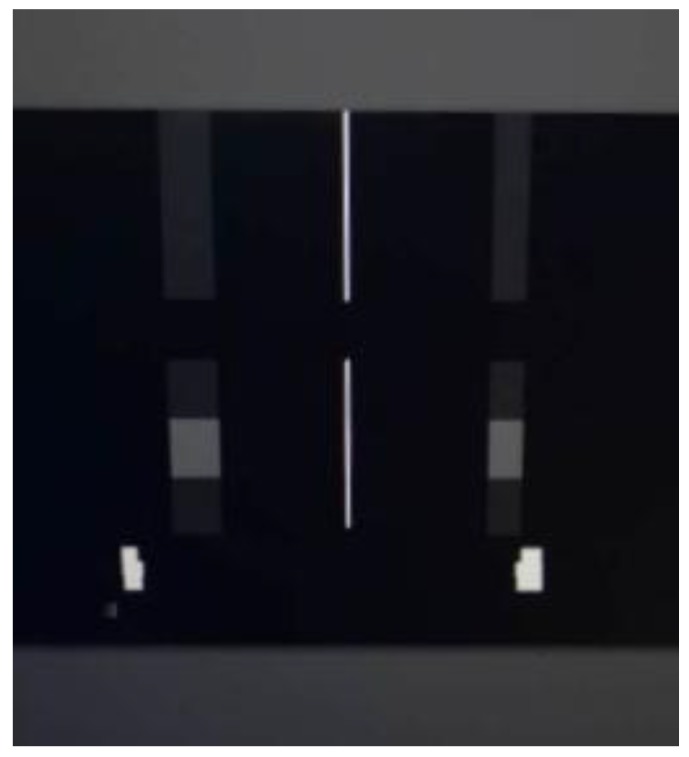
Image of the narrow line target taken by the camera.

The satellite was suspended to a horizontal state using a low-frequency suspension system for the purpose of offsetting the effect of gravity on the test results. Given that the first mode frequency of the satellite on orbit was 64 Hz, the suspension system was tuned to give a 4 Hz axial isolation frequency, which was an order of magnitude softer. Furthermore, the flywheel was started and worked steadily at a constant speed. The image was taken by camera, and the acceleration signal of each optical component was collected by the signal acquisition system with the sampling frequency set to 5020 Hz. From the above simulation, the influence ratio of the secondary mirror and image surface on image motion within 500 Hz was quite small. Note that the effective installation space of the secondary mirror and image surface was limited. Thus, the acceleration sensors were pasted on the back of the primary mirror and tertiary mirror. Figure 10 shows the distribution of three acceleration sensors pasted on the back of the primary mirror. To make the focal plane bright enough and reduce the diffraction intensity in the imaging test, the experiment was carried out with a narrow line target width of 13.75 um corresponding to 2.5 pixels on the focal plane. Figure 11 displays the image of the narrow line target taken by the camera.

### 4.2. Data Processing

Double integration was applied to infer displacement of the test points to obtain the output data of acceleration. By dividing the computation time into many short time periods, acceleration can be defined as follows [30]:

(3) $$\ddot{y}\left(t+\tau \right)=\ddot{y}\left(t\right)+\frac{\ddot{y}\left(t+\Delta t\right)-\ddot{y}\left(t\right)}{\Delta t}\tau$$

where Δt represents the short time period. The velocity and displacement can be calculated by integrating Equation (3).

(4) $$\int \ddot{y}\left(t+\tau \right)d\tau =\dot{y}\left(t+\tau \right)=\ddot{y}\left(t\right)\tau +\frac{\ddot{y}\left(t+\Delta t\right)-\ddot{y}\left(t\right)}{\Delta t}\;\frac{\tau^{2}}{2}+C$$

(5) $$\iint \ddot{y}\left(t+\tau \right)d\tau d\tau =y\left(t+\tau \right)=\ddot{y}\left(t\right)\frac{\tau^{2}}{2}+\frac{\ddot{y}\left(t+\Delta t\right)-\ddot{y}\left(t\right)}{\Delta t}\;\frac{\tau^{3}}{6}+C\tau +D$$

In case τ=0, $\dot{y}\left(t+\tau \right)=\dot{y}\left(t\right)$, and y(t+τ)=y(t), the constant can be calculated as:

(6) $$C=\dot{y}\left(t\right),\;D=y\left(t\right).$$

Substituting Equation (6) into Equations (4) and (5), the velocity and displacement at time t+Δt can be updated to give:

(7) $$\dot{y}\left(t+\Delta t\right)=\dot{y}\left(t\right)+\frac{\ddot{y}\left(t\right)+\ddot{y}\left(t+\Delta t\right)}{2}\Delta t$$

(8) $$y\left(t+\Delta t\right)=y\left(t\right)+\dot{y}\left(t\right)\Delta t+\frac{2\ddot{y}\left(t\right)+\ddot{y}\left(t+\Delta t\right)}{6}\Delta t^{2}$$

**Figure 12:**
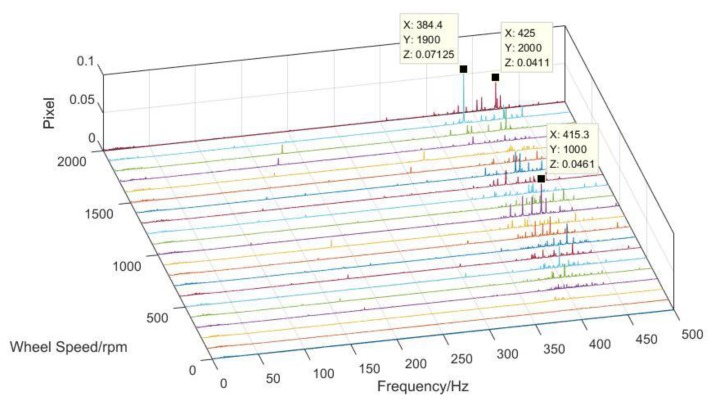
The pixel offset results of the test based on acceleration sensors in the frequency domain.

After obtaining the displacement of each test point on the optical component, the pixel offset of the whole optical system in the frequency domain can be calculated by using the same algorithm mentioned in Section 3.2, as shown in Figure 12.

**Figure 13:**
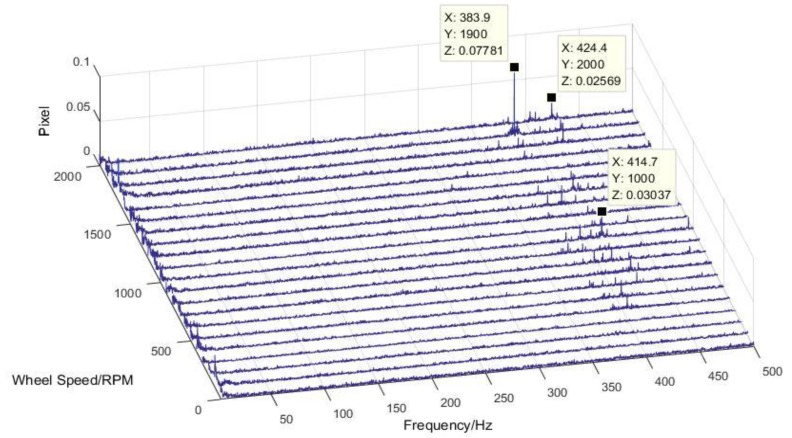
The pixel offset results of the imaging test in the frequency domain.

Data processing for the imaging test was undertaken in a MATLAB environment. For the sake of analyzing the vibration characteristics from the image, a grayscale threshold set at 90% of the maximum digital number was used to remove random noise. The narrow line was then extracted from the image, and the strip noise was removed by a smoothing filter. The centroid of each column was calculated using arithmetic mean [31,32,33]. The pixel offset in the time domain could, thus, be obtained. The pixel offset in the frequency domain at different speeds could be calculated by FFT, as shown in Figure 13.

### 4.3. Comparison of the Two Test Results and the Simulation

It can be seen from the comparison of the two test results that the results based on the acceleration sensor were in good agreement with that of the imaging test in regards to the amplitude of pixel offset, distribution of peak frequency, and harmonic order characteristics. The effect of microvibrations on image motion were mainly concentrated at 350–450 Hz, which was similar to the simulation prediction. However, analysis was performed with critical damping (nominal 0.1%) set as a constant value, which was different from actual damping in the ground test. Therefore, the amplitude of simulation results has a difference to the test results.

To better verify the correctness of the experimental scheme, three peaks in different speeds were extracted to compare the corresponding results of the imaging test. According to the comparison, the frequency and amplitude of the three peaks in the two tests were nearly identical. In light of the difference of inherent sampling frequencies set by the signal acquisition equipment, a slight error of 0.5 Hz was found after FFT. The maximum tested amplitude was 0.071 pixels, with a relative error of 8.4% in the imaging test results, indicating that influence ratio analyses for each optical component were effective. It can be inferred that the error could be reduced with a complete input signal from every optical component.

## 5. Conclusions

From the analysis of characteristic spectra in the test results, perturbation of the flywheel was mainly concentrated on the first harmonic order and at a high frequency (over 300 Hz). The amplitude was significantly greater in a high frequency, including 330 Hz and 400–450 Hz.

Based on the linear optical model, the optical amplification factor of each optical component can be calculated, which is an indispensable parameter for simulation and data processing of the test results.

The effect of microvibration on image motion can be effectively predicted by the simulation. From the simulation results, the influence on pixel offset was mainly concentrated at 330 Hz and 400–450 Hz, which showed good agreement with the tendencies in the test results. However, because critical damping (nominal 0.1%) was set as a constant value, the amplitude had a big gap compared to the test results.

Perturbation had a greater effect on the primary mirror and tertiary mirror in this opto-mechanical system when the pixel offset fell below 500 Hz, wherein the influence ratio of the two components was more than 98%.

The experiment based on the high-precision acceleration sensor showed high consistency with the results of the imaging test. The relative error of amplitude was less than 8.4%, and the error of frequency was 0.5 Hz. This new method has effectively improved the efficiency of jitter measurements, and it can be used to measure microvibrations with high precision.

## Figures and Tables

**Table 1:** The optical amplification factor of each optical component.

Unit Transition/Tilt of Optical Component	Image Motion of the Central Image Point on the Focal Plane (mm)							
	Primary Mirror		Secondary Mirror		Tertiary Mirror		Image Surface	
	*+X*	*+Y*	*+X*	*+Y*	*+X*	*+Y*	*+X*	*+Y*
Transition in +*X* direction/mm	0.98	0	−1.4	0	1.36	0	−1	0
Tilt about +*X* direction/°	0	−20.66	0	9.09	0	−13.46	0	0
Transition in +*Y* direction/mm	0	1	0	−1.33	0	1.35	0	−1
Tilt about +*Y* direction/°	21.17	0	−9.2	0	13.72	0	0	0
Transition in +*Z* direction/mm	0.3	0	−0.13	0	0.05	0	−0.05	0
Tilt about +*Z* direction/°	0	0	0	0	0	0	0	0
